# Using the human blood index to investigate host biting plasticity: a systematic review and meta-regression of the three major African malaria vectors

**DOI:** 10.1186/s12936-018-2632-7

**Published:** 2018-12-18

**Authors:** James Orsborne, Luis Furuya-Kanamori, Claire L. Jeffries, Mojca Kristan, Abdul Rahim Mohammed, Yaw A. Afrane, Kathleen O’Reilly, Eduardo Massad, Chris Drakeley, Thomas Walker, Laith Yakob

**Affiliations:** 1 Department of Disease Control, London School of Hygiene & Tropical Medicine, London, UK; 2 Department of Population Medicine, College of Medicine, Qatar University, Doha, Qatar; 3 Department of Medical Microbiology, College of Health Sciences, University of Ghana, Korle Bu, Accra, Ghana; 4 School of Applied Mathematics, Fundacao Getulio Vargas, Rio de Janeiro, Brazil; 5 Department of Immunology & Infection, London School of Hygiene & Tropical Medicine, London, UK

**Keywords:** Blood meal analysis, Host preference, Mosquito, Biting preference, Blood index

## Abstract

**Background:**

The proportion of mosquito blood-meals that are of human origin, referred to as the ‘human blood index’ or HBI, is a key determinant of malaria transmission.

**Methods:**

A systematic review was conducted followed by meta-regression of the HBI for the major African malaria vectors.

**Results:**

Evidence is presented for higher HBI among *Anopheles gambiae* (M/S forms and *Anopheles coluzzii*/*An. gambiae* sensu stricto are not distinguished for most studies and, therefore, combined) as well as *Anopheles funestus* when compared with *Anopheles arabiensis* (prevalence odds ratio adjusted for collection location [i.e. indoor or outdoor]: 1.62; 95% CI 1.09–2.42; 1.84; 95% CI 1.35–2.52, respectively). This finding is in keeping with the entomological literature which describes *An. arabiensis* to be more zoophagic than the other major African vectors. However, analysis also revealed that HBI was more associated with location of mosquito captures (R^2^ = 0.29) than with mosquito (sibling) species (R^2^ = 0.11).

**Conclusions:**

These findings call into question the appropriateness of current methods of assessing host preferences among disease vectors and have important implications for strategizing vector control.

**Electronic supplementary material:**

The online version of this article (10.1186/s12936-018-2632-7) contains supplementary material, which is available to authorized users.

## Background

Malaria is transmitted through mosquito bites, making the vectors’ choice of which blood-host species to bite a central component of malaria epidemiology and ecology. In Africa, the majority of infections are transmitted by *Anopheles gambiae* sensu stricto (s.s.), *Anopheles coluzzii*, *Anopheles funestus* and *Anopheles arabiensis*. Conventional wisdom indicates that the first three vectors are anthropophagic while the latter sibling species is more zoophagic. Levels of anthropophagy/zoophagy are typically assessed using PCR to identify the host species from blood-meals in field-caught mosquitoes, and are then quantified according to the human blood index (HBI), defined as the proportion of blood-meals that are of human origin [1]. Because two mosquito bites on a human are required to complete the malaria parasite’s life-cycle, HBI has an inflated impact on metrics of transmission such as the basic reproduction number, the vectorial capacity and the critical density of mosquitoes for sustained transmission [2].

However, the HBI should not be perceived to have a singular, fixed value; all major African malaria vectors have demonstrable plasticity in the host species that they bite [3–5]. It has long been recognized that the same mosquito population will often adjust its biting towards a more locally available host species [1, 6]. This has important implications for malaria control policy. For example, recent studies have observed that increased outdoor biting followed the distribution of insecticide-treated bed nets [7]. In such circumstances, vector control tools that operate effectively outdoors become a critical component for eliminating local malaria transmission. Unfortunately, the huge malaria burden reduction achieved in the years since 2000 has relied disproportionately on control tools operating indoors [8], and there are limited effective malaria-vector control options for outdoor use.

One technology that shows promise for targeting mosquitoes regardless of whether they bite indoors or outdoors involves the use of systemic insecticides—chemicals applied directly to blood-hosts to kill mosquitoes that take a blood meal. This technology arose from the observation that mosquito mortality was increased following the consumption of sugar-meals [9] or blood-meals [10] containing ivermectin—a drug used for onchocerciasis control. Drugs approved for veterinary use, such as fipronil, have subsequently been demonstrated to have similar impact when livestock are dosed orally, or when the chemical is applied topically [11]. More recently, systemic insecticides have had durations of their efficacy extended through dosing with higher concentrations [12], combined dosing with adjuvants [13], and with use of sustained-release devices [14]. The stage is set for progress in development and evaluation of ivermectin for vector control [15]. Therefore, arguably it has never been more important to understand the distribution of malaria-vector bites on alternative host species. Here, the current evidence is systematically reviewed and a meta-regression conducted to identify the factors associated with higher HBI in sub-Saharan Africa.

## Methods

Findings from the systematic review were reported following the PRISMA guidelines [16]. The inclusion and exclusion criteria are listed in Table 1 and advanced search terms were developed following initial manual literature searches and a basic PubMed search (Table 2). The purpose of the initial search was to identify keywords and synonyms. The authors agreed on the search terms and inclusion/exclusion criteria before the systematic search was performed. The Ovid database was used to search available MEDLINE and EMBASS literature from inception to February 2018. Books were excluded from all searches as well as articles not written in English. Results were retrieved and collated using Mendeley desktop reference manager.

**Table 1:** Inclusion and exclusion criteria for systematic review

Inclusion criteria	Exclusion criteria
Studies which used blood meal analysis (PCR, ELISA or precipitin tests) to report the HBI	Semi field studies, studies using baited traps or choice experiments to investigate host preference
Studies performed in sub-Saharan Africa	Entomological studies not specifically reporting the HBI
Studies reporting the HBI for individual mosquito species	Studies not reporting total number of mosquitoes caught
Reporting HBI for *Anopheles gambiae, Anopheles funestus complex* or *Anopheles arabiensis* mosquito species	Data points based on less than 50 blood-fed mosquitoes in total for target species
Studies reporting trapping methodology including location of traps (indoors or outdoors)

**Table 2:** Search strategy for systematic review

Ovid MEDLINE^®^ Database Human blood index OR HBI OR host preference OR trophic preference OR blood meal preference OR blood host preference OR blood meal OR blood meal analysis OR blood-meal analysis OR blood meal source OR host blood OR host blood meal OR blood meal identification [multiple posting = MeSH subject heading word, abstract, title, original title, text word (title, abstract), key word heading, name of substance, key word heading word, protocol supplementary concept word, synonym] AND Anopheles OR Anopheles arabiensis OR Anopheles gambiae OR Anopheles funestus [multiple posting = MeSH subject heading word, abstract, title, original title, text word (title, abstract), key word heading, name of substance, key word heading word, protocol supplementary concept word, synonym]	

After eliminating duplications, abstracts for all publications retrieved were reviewed for relevance. Full-text reviews were then conducted on all articles to decide on its inclusion in accordance with the pre-specified inclusion and exclusion criteria. If the inclusion criteria were satisfied the estimated human blood index (HBI) reported was retrieved. Other variables that could have a significant effect on the reported HBI were also retrieved. These variables included (sibling) species (complex), trapping location (indoors, outdoors or both), trap type(s) used and total number of mosquitoes collected. The primary effect measure of interest was the HBI.

The double arcsine square root transformed HBI (expressed as a proportion of all blood-meals) was used to stabilize the variance across the studies [17] and then back transformed for ease of interpretation. A linear model was performed on all eligible studies to gain additional insight into the effect of trapping location and *Anopheles* species on the proportion of HBI. The linear model was fit using the HBI (proportion) as the response variable weighted by the inverse of each study’s variance to allow the observations with the least variance to provide the most information to the model, and using robust error variances. All tests were two-tailed and a p-value < 0.05 was deemed statistically significant. Inverse variance weights were obtained using MetaXL (version 5.3, EpiGear Int Pty Ltd; Sunrise Beach, Australia) and the regression models were run using Stata MP (version 14, Stata Corp, College Station, TX, USA).

## Results

**Fig. 1:**
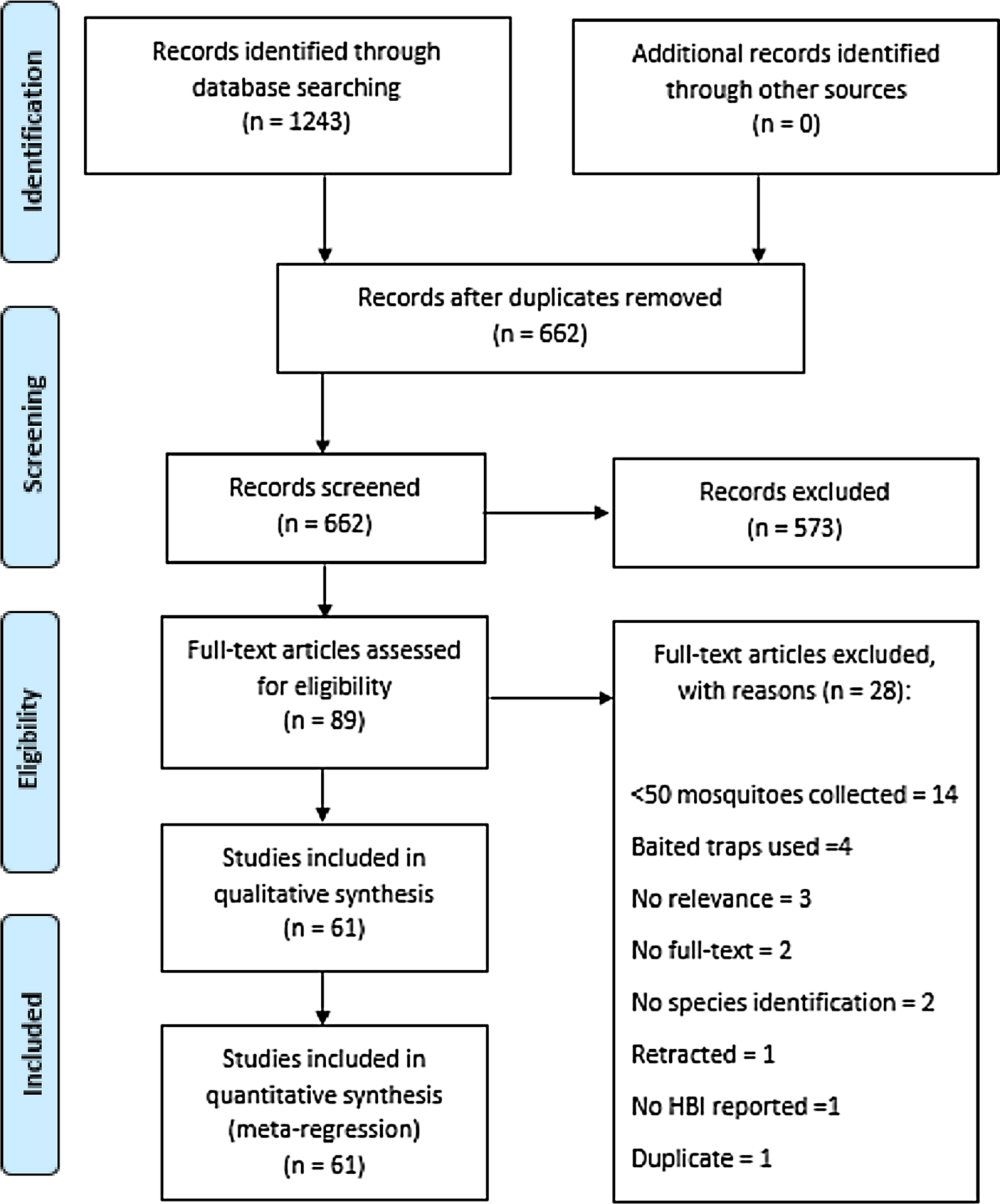
PRISMA flow diagram of search phases with numbers of studies included/excluded at each stage

The search identified 1243 potentially relevant studies. After collating these results and reviewing all abstracts, 662 studies were deemed relevant. All full text articles were retrieved, reviewed for relevance and reviewed against all inclusion and exclusion criteria. Sixty-one studies resulting in 166 data points fulfilled all criteria and where included in the analysis. Reasons for exclusion at full text stage included inadequate number (fewer than 50) of mosquitoes collected (n = 14) and the use of host-biased trapping methodologies (n = 4) (Fig. 1).

**Fig. 2:**
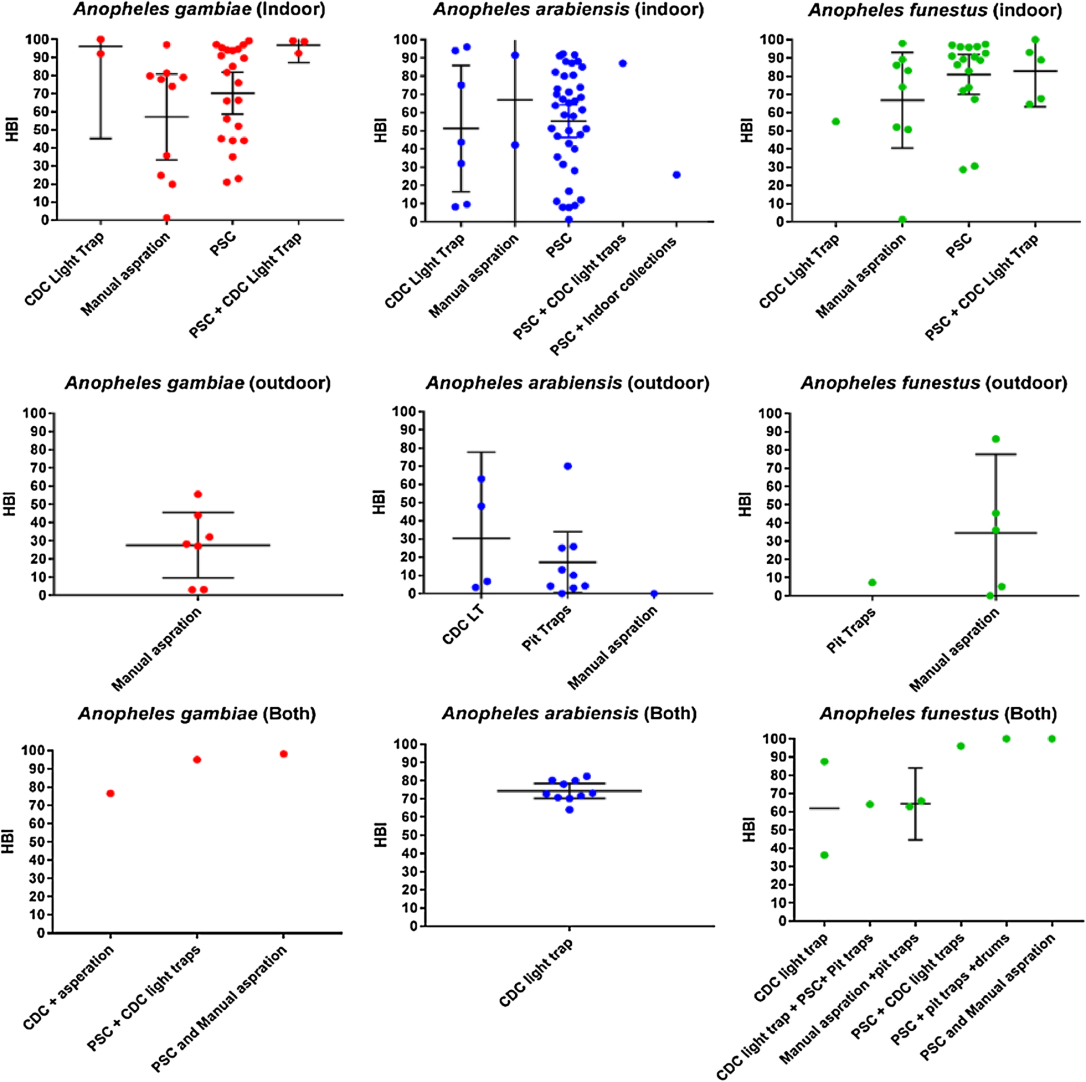
Reported mean HBI (+ 95% CIs) for individual collection methods when sampling from indoor, outdoor or both (indoor and outdoor) environments for *An. gambiae, An. arabiensis* and *An. funestus*

Multiple collection methodologies were identified from the eligible studies. The methodology used was governed by the collection location targeted (indoors or outdoors). Indoor collections were the most widely used (n = 118) with pyrethroid spray catch (PSC) the most commonly used methodology (n = 78). Other collection methods included manual indoor collections (n = 20) and the use of CDC light traps within the household (n = 10). Outdoor collections represented 27 of the total data points extracted with manual collection of mosquitoes being the most common collection method (n = 13). Pit traps (n = 10) and CDC light traps (n = 4) were also an effective collection method. Studies collecting from both indoor and outdoor environments consisted of 21 data points. These studies used a variety of different methods; many used a combination of the most effective indoor and outdoor collection methods. CDC light traps were the most common (n = 12) followed by other combinations of indoor and outdoor methods; CDC light trap plus PSC (n = 2) and pit traps and manual indoor collections (n = 2) (Table 3 and Additional file 1). Collection methods had no significant effect (p > 0.05) on the reported HBI when comparing the mean HBI produced by each collection methodology within its respective collection areas (indoor and outdoor) for *An. gambiae, An. arabiensis* and the *An. funestus* species complex (Fig. 2). It should be noted that due to the variety of different methods used and therefore sparsity of data for each methodology within the “both” categories, a meaningful comparison could not be made.

**Table 3:** Data points extracted from eligible studies for each collection location and trapping methodology

Species	Collection location			Methodology								
				Indoor			Outdoor			Both (indoor + outdoor)		
	Indoor	Outdoor	Both	Pyrethroid spray catch (PSC)	Manual aspiration	CDC light trap	Manual aspiration	CDC light trap	Pit traps	CDC light trap + PSC	CDC light trap	Others
*Anopheles gambiae*	37	7	3	22	10	2	7	0	0	1	0	2
*Anopheles arabiensis*	50	14	10	39	2	7	1	4	9	0	10	0
*Anopheles funestus* s.l.	31	6	8	17	8	1	5	0	1	1	2	5

The top three methodologies based on number of data points extracted are displayed here

Meta-regression of the data compiled from the 166 data points demonstrated a significantly higher proportion of blood-meals were of human origin (the human blood index, ‘HBI’) among *An*. *funestus* (prevalence odds ratios [POR] of 1.84 (95% CI 1.35–2.52, p < 0.001) and *An*. *gambiae* (POR of 1.62, 95% CI 1.09–2.52, p = 0.02) compared to *An*. *arabiensis*. The majority of studies including details of *An*. *gambiae* did not specify whether they were M or S forms (or, in more modern nomenclature, *An. coluzzii* or *An. gambiae* s.s.), so these were combined. For all three groups, a significantly higher HBI was found from indoor mosquito collections (POR of 2.74, 95% CI 2.00–3.75, p < 0.001) as well as combined indoor and outdoor collections (POR of 4.20, 95% CI 3.13–5.62, p < 0.001) versus outdoor only collections. *Anopheles* species was not found to be an interaction term for location collection and HBI, indicating that all species follow a similar trend regarding their preferred location for biting humans. The results also revealed that trapping location (R^2^ of 0.29) had a larger impact on the blood-meal host species than mosquito species (or species complex) (R^2^ of 0.11) and that this difference was statistically significant (p < 0.01 resulting from an F-test comparing both univariable models) (Table 4).

**Table 4:** Predictors of human blood index: univariable and multivariable regression models

Predictors	Univariable		Multivariable	
	POR (95% CI)	R^2^	POR (95% CI)	R^2^
Anopheles species		0.11		0.40
*An. arabiensis*	1.00		1.00	
*An. gambiae*^a^	1.50 (0.95–2.36)		1.62 (1.09–2.42)	
*An. funestus*	1.95 (1.34–2.85)		1.84 (1.35–2.52)	
Location		0.29		
Outdoor only	1.00		1.00	
Indoor only	2.83 (2.04–3.93)		2.74 (2.00–3.75)	
Both	3.98 (3.14–5.06)		4.20 (3.13–5.62)	

*POR* prevalence odds ratio

^a^Most studies did not specify M/S form of *An*. *gambiae* (and pre-dated the renaming of these forms as *An. coluzzii* and *An. gambiae* s.s., respectively)

## Discussion

Control of vector-borne diseases is largely, often entirely, dependent on vector control. For malaria, vector control is achieved primarily through targeting mosquitoes that are host-seeking [8]. The major African malaria vectors, *An. gambiae* s.s., *An. coluzzii* and *An*. *funestus*, are regularly cited as paragons of anthropophagy, and any non-human biting exhibited by these species has historically been ignored when strategizing control. Here, their biting behaviour was systematically reviewed and clearly demonstrated that the difference in their host choice compared with the zoophilic vector *An*. *arabiensis* was dwarfed by the difference found when comparing indoor with outdoor collections. In other words, *where* the mosquito was collected was substantially and significantly more influential on host choice than *which* mosquito species was collected.

This raises an important question: where should vectors be collected from in order to provide the most useful HBI estimates? Results indicate that a single HBI for a given location risks presenting quite a biased estimate for local vector biting behaviour. A standardized HBI accounting for both indoor and outdoor behaviours would probably constitute an invalid metric because of the increased difficulty posed by collecting blood-fed mosquitoes outdoors i.e., tools are lacking for the estimation of indoor *versus* outdoor mosquito numbers with any confidence. Therefore, current best practice should be to present both estimates for an indoor HBI and an outdoor HBI. Longitudinal assessments initiated before rolling out control tools, and followed up over the time course of the programme would provide a valuable source of information. For example, these would determine the timeframe across which LLIN-derived exophagy [7], as well as zoophagy [18] occurs, as well as provide unbiased estimates of the magnitude of effect. These entomological data would also be able to inform on whether there is a reversion to behavioural norm after a certain period post-distribution, and the rate at which this occurred.

Better data on this behaviour and its temporality will do much more than inform a fundamental aspect of mosquito ecology: it will have considerable ramifications pertaining to malaria control. For example, if significantly reduced HBI is detected immediately following the distribution of LLINs, this may present an excellent opportunity to synergize bed nets with systemic insecticide-treated livestock. Saul [19] described the potential for zooprophylaxis to switch into zoopotentiation if the availability of alternative blood meals increases mosquito survival more than counters the impact of diverting feeds. This risk could be reduced or eliminated with systemic insecticidal dosing that is judiciously timed with LLIN roll-out. Mathematical models already exist for optimal systemic insecticide deployment [20] including its integration with LLINs [21]. These could immediately be capitalized upon once the temporal HBI data became available.

One further, important unknown pertaining to HBI is the spatial scale across which within-mosquito population plasticity occurs. Over 50 years ago, Garrett-Jones described differing HBI estimates for mosquitoes collected from proximal locations [1]. Given the current concerns over altered biting behaviour potentially compromising recent gains in malaria burden reduction [22], a fuller comprehension of the scale and magnitude of this variability is timely. A recent study conducted in southern Ghana describes the successful piloting of a novel experimental design to address exactly this phenomenon [23]. It demonstrated that statistically significant alteration in host choice for *An. coluzzii* was detectable over a range of 250 m [23]. Heterogeneity in mosquito biting rates has been demonstrated to be key to malaria transmission, first by theoretical work [24], but more recently with empirical studies using genotyping of blood-meals [25]. Future modelling frameworks will need to account for this additional form of village-level heterogeneity in biting behaviour.

## Conclusion

Results demonstrate that where mosquitoes are collected from (indoors *versus* outdoors) is significantly more associated with the HBI than which of the major African malaria-vector mosquito (sibling) species is collected. Some of the more important consequences to disease control of this behaviour are described. Some new theoretical and empirical developments that may improve both HBI assessment and how this metric can inform malaria control optimisation are discussed.

## Additional file

**Additional file 1.** All eligible studies (and corresponding data points) retrieved from systematic search.
