# Supplementary material for: Using the human blood index to investigate host biting plasticity: a systematic review and meta-regression of the three major African malaria vectors
Source: Malar J. 2018 Dec 18;17:479. doi: 10.1186/s12936-018-2632-7 (PMC6299493; doi:10.1186/s12936-018-2632-7)
Supplement: Supplementary file 1 — Additional file 1. All eligible studies (and corresponding data points) retrieved from systematic search. [file 12936_2018_2632_MOESM1_ESM.docx]

**Additional file 1: All eligible studies (and corresponding data points) retrieved from systematic search**

| **Lead Author** | **Year Published** | **Reference** | **Location** | **Species** | **collection area (indoor/outdoor/both)** | **collection method** | **human blood** | **Total number of bloodfeds analysed/caught** |
| --- | --- | --- | --- | --- | --- | --- | --- | --- |
| Das | 2017 | Beyond the entomological inoculation rate: characterizing multiple blood feeding behaviour and *Plasmodium falciparum* multiplicity of infection in *Anopheles* mosquitoes in northern Zambia | Zambia | *Anopheles funestus complex* | Both | PSC + CDC | 426 | 444 |
| Das | 2017 | Beyond the entomological inoculation rate: characterizing multiple blood feeding behaviour and *Plasmodium falciparum* multiplicity of infection in *Anopheles* mosquitoes in northern Zambia | Zambia | *Anopheles gambiae* | Both | PSC + CDC | 95 | 100 |
| Ogola | 2017 | Composition of *Anopheles* mosquitoes, their blood-meal hosts, and *Plasmodium falciparum* infection rates in three islands with disparate bed net coverage in Lake Victoria, Kenya. | Kenya | *Anopheles gambiae* | Both | CDC + outdoor manual collections | 236 | 310 |
| Degefa | 2017 | Indoor and outdoor malaria vector surveillance in western Kenya: implications for better understanding of residual transmission | Kenya | *Anopheles arabiensis* | Indoors | CDC | 10 | 122 |
| Degefa | 2017 | Indoor and outdoor malaria vector surveillance in western Kenya: implications for better understanding of residual transmission | Kenya | *Anopheles arabiensis* | Indoors | PSC | 1 | 165 |
| Degefa | 2017 | Indoor and outdoor malaria vector surveillance in western Kenya: implications for better understanding of residual transmission | Kenya | *Anopheles arabiensis* | Outdoors | CDC | 2 | 59 |
| Degefa | 2017 | Indoor and outdoor malaria vector surveillance in western Kenya: implications for better understanding of residual transmission | Kenya | *Anopheles arabiensis* | Outdoors | Pit traps | 208 | 298 |
| Kibret | 2017 | Malaria impact of large dams at different eco-epidemiological settings in Ethiopia | Ethopia | *Anopheles arabiensis* | Both | CDC | 761 | 924 |
| Kibret | 2017 | Malaria impact of large dams at different eco-epidemiological settings in Ethiopia | Ethopia | *Anopheles arabiensis* | Both | CDC | 202 | 278 |
| Kibret | 2017 | Malaria impact of large dams at different eco-epidemiological settings in Ethiopia | Ethopia | *Anopheles arabiensis* | Both | CDC | 277 | 392 |
| Kibret | 2017 | Malaria impact of large dams at different eco-epidemiological settings in Ethiopia | Ethopia | *Anopheles arabiensis* | Both | CDC | 117 | 168 |
| Kibret | 2017 | Malaria impact of large dams at different eco-epidemiological settings in Ethiopia | Ethopia | *Anopheles funestus complex* | Both | CDC | 272 | 311 |
| Kabula | 2016 | A significant association between deltamethrin resistance, *Plasmodium falciparum*infection and the Vgsc-1014S resistance mutation in *Anopheles gambiae* highlights the epidemiological importance of resistance markers | Tanzania | *Anopheles gambiae* | Indoors | PSC | 548 | 575 |
| Kabula | 2016 | A significant association between deltamethrin resistance, *Plasmodium falciparum* infection and the Vgsc-1014S resistance mutation in *Anopheles gambiae* highlights the epidemiological importance of resistance markers | Tanzania | *Anopheles arabiensis* | Indoors | PSC | 409 | 575 |
| Sande | 2016 | Biting behaviour of *Anopheles funestus* populations in Mutare and Mutasa districts, Manicaland province, Zimbabwe: Implications for the malaria control programme | Zimbabwe | *Anopheles funestus complex* | Both | CDC,PSC and Pit traps | 174 | 272 |
| Chirebvu | 2016 | Characterization of an indoor-resting population of *Anopheles arabiensis*(Diptera: Culicidae) and the implications on malaria transmission in Tubu village in Okavango Subdistrict, Botswana. | Botswana | *Anopheles arabiensis* | Indoors | PSC + Indoor manual collections | 35 | 139 |
| Lekweiry | 2016 | Circumsporozoite protein rates, blood-feeding pattern and frequency of knockdown resistance mutations in *Anopheles* spp. in two ecological zones of Mauritania | Mauritania | *Anopheles arabiensis* | Indoors | PSC | 46 | 80 |
| Ndiath | 2016 | Composition and genetics of malaria vector populations in the Central African Republic | Central African Republic | *Anopheles gambiae* | Indoors | PSC | 121 | 149 |
| Lozano-Fuentes | 2016 | Evaluation of a topical formulation of eprinomectin against *Anopheles arabiensis* when administered to Zebu cattle (*Bos indicus*) under field conditions | kenya | *Anopheles arabiensis* | Indoors | PSC | 10 | 131 |
| Lozano-Fuentes | 2016 | Evaluation of a topical formulation of eprinomectin against *Anopheles arabiensis* when administered to Zebu cattle (*Bos indicus*) under field conditions | kenya | *Anopheles gambiae* | Indoors | PSC | 77 | 91 |
| Mosqueira | 2015 | Pilot study on the combination of an organophosphate-based insecticide paint and pyrethroid-treated long lasting nets against pyrethroid resistant malaria vectors in Burkina Faso. | Burkina Faso | *Anopheles gambiae* | Indoors | Indoor manual collection | 34 | 141 |
| Mosqueira | 2015 | Pilot study on the combination of an organophosphate-based insecticide paint and pyrethroid-treated long lasting nets against pyrethroid resistant malaria vectors in Burkina Faso. | Burkina Faso | *Anopheles gambiae* | Indoors | Indoor manual collection | 51 | 143 |
| Mosqueira | 2015 | Pilot study on the combination of an organophosphate-based insecticide paint and pyrethroid-treated long-lasting nets against pyrethroid resistant malaria vectors in Burkina Faso. | Burkina Faso | *Anopheles gambiae* | Indoors | Indoor manual collection | 28 | 141 |
| Das | 2015 | Underestimation of foraging behaviour by standard field methods in malaria vector mosquitoes in southern Africa. | Zambia and Zimbabwe | *Anopheles arabiensis* | Indoors | PSC + CDC | 559 | 643 |
| Das | 2015 | Underestimation of foraging behaviour by standard field methods in malaria vector mosquitoes in southern Africa. | Zambia and Zimbabwe | *Anopheles funestus complex* | Indoors | PSC + CDC | 343 | 343 |
| Das | 2015 | Underestimation of foraging behaviour by standard field methods in malaria vector mosquitoes in southern Africa. | Zambia and Zimbabwe | *Anopheles funestus complex* | Indoors | PSC + CDC | 78 | 84 |
| Massebo | 2015 | Zoophagic behaviour of anopheline mosquitoes in southwest Ethiopia: opportunity for malaria vector control | Ethopia | *Anopheles arabiensis* | Indoors | CDC | 93 | 988 |
| Massebo | 2015 | Zoophagic behaviour of anopheline mosquitoes in southwest Ethiopia: opportunity for malaria vector control | Ethopia | *Anopheles arabiensis* | Indoors | PSC | 59 | 352 |
| Massebo | 2015 | Zoophagic behaviour of anopheline mosquitoes in southwest Ethiopia: opportunity for malaria vector control | Ethopia | *Anopheles arabiensis* | Outdoors | Pit traps | 26 | 894 |
| Guelbeogo | 2014 | Behavioural divergence of sympatric *Anopheles funestus*populations in Burkina Faso. | Burkina Faso | *Anopheles funestus complex* | Indoors | PSC | 211 | 221 |
| Guelbeogo | 2014 | Behavioural divergence of sympatric *Anopheles funestus*populations in Burkina Faso. | Burkina Faso | *Anopheles funestus complex* | Indoors | PSC | 242 | 272 |
| Guelbeogo | 2014 | Behavioural divergence of sympatric *Anopheles funestus*populations in Burkina Faso. | Burkina Faso | *Anopheles funestus complex* | Outdoors | Pit traps | 38 | 529 |
| Sougoufara | 2014 | Biting by *Anopheles funestus* in broad daylight after use of long-lasting insecticidal nets: a new challenge to malaria elimination | Senigal | *Anopheles funestus complex* | Indoors | PSC | 61 | 84 |
| Antonio-Nkondjio | 2014 | High malaria transmission intensity in a village close to Yaoundé, the capital city of Cameroon. | Cameroon | *Anopheles funestus complex* | Both | PSC + pit traps +drums | 299 | 299 |
| Kibret | 2014 | Increased malaria transmission around irrigation schemes in Ethiopia and the potential of canal water management for malaria vector control | Ethopia | *Anopheles funestus complex* | Both | CDC | 20 | 58 |
| Kibret | 2014 | Increased malaria transmission around irrigation schemes in Ethiopia and the potential of canal water management for malaria vector control | Ethopia | *Anopheles arabiensis* | Both | CDC | 1680 | 2101 |
| Kibret | 2014 | Increased malaria transmission around irrigation schemes in Ethiopia and the potential of canal water management for malaria vector control | Ethopia | *Anopheles arabiensis* | Both | CDC | 171 | 234 |
| McCann | 2014 | Reemergence of *Anopheles funestus* as a vector of *Plasmodium falciparum* in Western Kenya after long-term implementation of insecticide-treated bed nets | Kenya | *Anopheles funestus complex* | Indoors | PSC | 697 | 715 |
| McCann | 2014 | Reemergence of *Anopheles funestus* as a Vector of *Plasmodium falciparum* in Western Kenya after long-term implementation of insecticide-treated bed nets | Kenya | *Anopheles arabiensis* | Indoors | PSC | 58 | 115 |
| McCann | 2014 | Reemergence of *Anopheles funestus* as a vector of *Plasmodium falciparum* in Western Kenya after long-term implementation of insecticide-treated bed nets | Kenya | *Anopheles gambiae* | Indoors | PSC | 51 | 55 |
| McCann | 2014 | Reemergence of *Anopheles funestus* as a vector of *Plasmodium falciparum* in Western Kenya after long-term implementation of insecticide-treated bed nets | Kenya | *Anopheles arabiensis* | Indoors | PSC | 25 | 73 |
| Massebo | 2013 | Blood meal origins and insecticide susceptibility of *Anopheles arabiensis* from Chano in South-West Ethiopia | Ethopia | *Anopheles arabiensis* | Indoors | CDC | 741 | 988 |
| Massebo | 2013 | Blood meal origins and insecticide susceptibility of *Anopheles arabiensis* from Chano in South-West Ethiopia | Ethopia | *Anopheles arabiensis* | Indoors | PSC | 204 | 352 |
| Massebo | 2013 | Blood meal origins and insecticide susceptibility of *Anopheles arabiensis* from Chano in South-West Ethiopia | Ethopia | *Anopheles arabiensis* | Outdoors | Pit traps | 116 | 894 |
| Animut | 2013 | Blood meal sources and entomological inoculation rates of anophelines along a highland altitudinal transect in south-central Ethiopia | Ethopia | *Anopheles arabiensis* | Indoors | CDC | 135 | 422 |
| Animut | 2013 | Blood meal sources and entomological inoculation rates of anophelines along a highland altitudinal transect in south-central Ethiopia | Ethopia | *Anopheles arabiensis* | Outdoors | PSC | 227 | 723 |
| Animut | 2013 | Blood meal sources and entomological inoculation rates of anophelines along a highland altitudinal transect in south-central Ethiopia | Ethopia | *Anopheles arabiensis* | Indoors | CDC | 27 | 64 |
| Animut | 2013 | Blood meal sources and entomological inoculation rates of anophelines along a highland altitudinal transect in south-central Ethiopia | Ethopia | *Anopheles arabiensis* | Outdoors | PSC | 32 | 114 |
| Dadzie | 2013 | Role of species composition in malaria transmission by the *Anopheles funestus* group (Diptera: Culicidae) in Ghana | Ghana | *Anopheles funestus complex* | Indoors | PSC | 80 | 89 |
| Dadzie | 2013 | Role of species composition in malaria transmission by the *Anopheles funestus* group (Diptera: Culicidae) in Ghana | Ghana | *Anopheles funestus complex* | Indoors | PSC | 52 | 64 |
| Dadzie | 2013 | Role of species composition in malaria transmission by the *Anopheles funestus* group (Diptera: Culicidae) in Ghana | Ghana | *Anopheles funestus complex* | Indoors | PSC | 73 | 76 |
| Obala | 2012 | *Anopheles gambiae* and *Anopheles arabiensis* population densities and infectivity in Kopere village, Western Kenya | Kenya | *Anopheles arabiensis* | Indoors | PSC | 59 | 68 |
| Obala | 2012 | *Anopheles gambiae* and *Anopheles arabiensis* population densities and infectivity in Kopere village, Western Kenya | Kenya | *Anopheles gambiae* | Indoors | PSC | 198 | 205 |
| Mzilahowa | 2012 | Entomological indices of malaria transmission in Chikhwawa district, Southern Malawi | Malawi | *Anopheles funestus complex* | Indoors | PSC | 286 | 297 |
| Mzilahowa | 2012 | Entomological indices of malaria transmission in Chikhwawa district, Southern Malawi | Malawi | *Anopheles gambiae* | Indoors | PSC | 244 | 246 |
| Mzilahowa | 2012 | Entomological indices of malaria transmission in Chikhwawa district, Southern Malawi | Malawi | *Anopheles arabiensis* | Indoors | PSC | 289 | 340 |
| Kibret | 2012 | How does an Ethiopian dam increase malaria? Entomological determinants around the Koka reservoir. | Ethopia | *Anopheles arabiensis* | Both | CDC | 148 | 208 |
| Kibret | 2012 | How does an Ethiopian dam increase malaria? Entomological determinants around the Koka reservoir. | Ethopia | *Anopheles arabiensis* | Both | CDC | 89 | 111 |
| Kibret | 2012 | How does an Ethiopian dam increase malaria? Entomological determinants around the Koka reservoir. | Ethopia | *Anopheles arabiensis* | Both | CDC | 56 | 89 |
| Kawada | 2012 | Reconsideration of *Anopheles rivulorum* as a vector of *Plasmodium falciparum* in western Kenya: some evidence from biting time, blood preference, sporozoite positive rate, and pyrethroid resistance | Kenya | *Anopheles funestus complex* | Indoors | Indoor manual collection | 34 | 69 |
| Tanga | 2011 | Daily survival and human blood index of major malaria vectors associated with oil palm cultivation in Cameroon and their role in malaria transmission. | Cameroon | *Anopheles funestus complex* | Indoors | PSC | 237 | 245 |
| Himeidan | 2011 | Pattern of malaria transmission along the Rahad River basin, Eastern Sudan | Sudan | *Anopheles arabiensis* | Indoors | PSC | 176 | 219 |
| Himeidan | 2011 | Pattern of malaria transmission along the Rahad River basin, Eastern Sudan | Sudan | *Anopheles arabiensis* | Indoors | PSC | 68 | 102 |
| Himeidan | 2011 | Pattern of malaria transmission along the Rahad River basin, Eastern Sudan | Sudan | *Anopheles arabiensis* | Indoors | PSC | 37 | 58 |
| Himeidan | 2011 | Pattern of malaria transmission along the Rahad River basin, Eastern Sudan | Sudan | *Anopheles arabiensis* | Indoors | PSC | 361 | 394 |
| Himeidan | 2011 | Pattern of malaria transmission along the Rahad River basin, Eastern Sudan | Sudan | *Anopheles arabiensis* | Indoors | PSC | 95 | 119 |
| Himeidan | 2011 | Pattern of malaria transmission along the Rahad River basin, Eastern Sudan | Sudan | *Anopheles arabiensis* | Indoors | PSC | 39 | 64 |
| Himeidan | 2011 | Pattern of malaria transmission along the Rahad River basin, Eastern Sudan | Sudan | *Anopheles arabiensis* | Indoors | PSC | 272 | 331 |
| Mala | 2011 | *Plasmodium falciparum* transmission and aridity: a Kenyan experience from the dry lands of Baringo and its implications for *Anopheles arabiensis* control | Kenya | *Anopheles arabiensis* | Outdoors | CDC | 55 | 88 |
| Mala | 2011 | *Plasmodium falciparum* transmission and aridity: a Kenyan experience from the dry lands of Baringo and its implications for *Anopheles arabiensis* control | Kenya | *Anopheles arabiensis* | Indoors | PSC | 58 | 136 |
| Mala | 2011 | *Plasmodium falciparum* transmission and aridity: a Kenyan experience from the dry lands of Baringo and its implications for *Anopheles arabiensis* control | Kenya | *Anopheles arabiensis* | Outdoors | CDC | 71 | 149 |
| Fornadel | 2010 | Analysis of *Anopheles arabiensis* blood feeding behaviour in Southern Zambia during the two years after introduction of insecticide-treated bed nets | Zambia | *Anopheles arabiensis* | Indoors | CDC | 220 | 235 |
| Fornadel | 2010 | Analysis of *Anopheles arabiensis* blood feeding behaviour in Southern Zambia during the two years after introduction of insecticide-treated bed nets | Zambia | *Anopheles arabiensis* | Indoors | CDC | 223 | 233 |
| Tchuinkam | 2010 | Bionomics of Anopheline species and malaria transmission dynamics along an altitudinal transect in Western Cameroon. | Cameroon | *Anopheles gambiae* | Indoors | PSC | 269 | 278 |
| Tchuinkam | 2010 | Bionomics of Anopheline species and malaria transmission dynamics along an altitudinal transect in Western Cameroon. | Cameroon | *Anopheles gambiae* | Indoors | PSC | 68 | 77 |
| Tchuinkam | 2010 | Bionomics of Anopheline species and malaria transmission dynamics along an altitudinal transect in Western Cameroon. | Cameroon | *Anopheles gambiae* | Indoors | PSC | 347 | 371 |
| Tanga | 2010 | Climate change and altitudinal structuring of malaria vectors in south-western Cameroon: their relation to malaria transmission | Cameroon | *Anopheles gambiae* | Both | PSC and outdoor manual collections | 109 | 112 |
| Tanga | 2010 | Climate change and altitudinal structuring of malaria vectors in south-western Cameroon: their relation to malaria transmission | Cameroon | *Anopheles funestus complex* | Both | PSC and outdoor manual collections | 63 | 63 |
| Adeleke | 2010 | Population dynamics of indoor sampled mosquitoes and their implication in disease transmission in Abeokuta, south-western Nigeria | Nigeria | *Anopheles gambiae* | Indoors | CDC | 225 | 225 |
| Kibret | 2010 | The impact of a small-scale irrigation scheme on malaria transmission in Ziway area, Central Ethiopia. | Ethopia | *Anopheles arabiensis* | Both | CDC | 93 | 120 |
| Kasili | 2009 | Entomological assessment of the potential for malaria transmission in Kibera slum of Nairobi, Kenya | Kenya | *Anopheles gambiae* | Indoors | Indoor manual collection | 77 | 80 |
| Kerah-Hinzoumbé | 2009 | Malaria vectors and transmission dynamics in Goulmoun, a rural city in south-western Chad | Chad | *Anopheles arabiensis* | Indoors | PSC | 92 | 144 |
| Kerah-Hinzoumbé | 2009 | Malaria vectors and transmission dynamics in Goulmoun, a rural city in south-western Chad | Chad | *Anopheles funestus complex* | Indoors | PSC | 48 | 53 |
| Caputo | 2008 | *Anopheles gambiae*complex along The Gambia river, with particular reference to the molecular forms of *An. gambiae s.s.* | Gambia | *Anopheles gambiae* | Indoors | PSC | 36 | 56 |
| Caputo | 2008 | *Anopheles gambiae*complex along The Gambia river, with particular reference to the molecular forms of *An. gambiae s.s.* | Gambia | *Anopheles gambiae* | Indoors | PSC | 71 | 158 |
| Caputo | 2008 | *Anopheles gambiae* complex along The Gambia river, with particular reference to the molecular forms of *An. gambiae s.s.* | Gambia | *Anopheles gambiae* | Indoors | PSC | 16 | 73 |
| Caputo | 2008 | *Anopheles gambiae*complex along The Gambia river, with particular reference to the molecular forms of *An. gambiae s.s.* | Gambia | *Anopheles gambiae* | Indoors | PSC | 36 | 82 |
| Caputo | 2008 | *Anopheles gambiae* complex along The Gambia river, with particular reference to the molecular forms of *An. gambiae s.s.* | Gambia | *Anopheles gambiae* | Indoors | PSC | 29 | 68 |
| Caputo | 2008 | *Anopheles gambiae*complex along The Gambia river, with particular reference to the molecular forms of *An. gambiae s.s.* | Gambia | *Anopheles gambiae* | Indoors | PSC | 18 | 89 |
| Caputo | 2008 | *Anopheles gambiae*complex along The Gambia river, with particular reference to the molecular forms of *An. gambiae s.s.* | Gambia | *Anopheles gambiae* | Indoors | PSC | 51 | 68 |
| Caputo | 2008 | *Anopheles gambiae*complex along The Gambia river, with particular reference to the molecular forms of *An. gambiae s.s.* | Gambia | *Anopheles gambiae* | Indoors | PSC | 62 | 179 |
| Muturi | 2008 | Effect of rice cultivation on malaria transmission in Central Kenya | Kenya | *Anopheles arabiensis* | Indoors | PSC | 73 | 812 |
| Muturi | 2008 | Effect of rice cultivation on malaria transmission in Central Kenya | Kenya | *Anopheles arabiensis* | Indoors | PSC | 40 | 334 |
| Muturi | 2008 | Effect of rice cultivation on malaria transmission in Central Kenya | Kenya | *Anopheles arabiensis* | Indoors | PSC | 65 | 131 |
| Muturi | 2008 | Effect of rice cultivation on malaria transmission in Central Kenya | Kenya | *Anopheles funestus complex* | Indoors | PSC | 46 | 65 |
| Fornadel | 2008 | Increased endophily by the malaria vector *Anopheles arabiensis* in Southern Zambia and identification of digested blood meals | Zambia | *Anopheles arabiensis* | Indoors | PSC | 252 | 289 |
| Abdalla | 2008 | Insecticide susceptibility and vector status of natural populations of *Anopheles arabiensis* from Sudan | Sudan | *Anopheles arabiensis* | Indoors | PSC | 273 | 310 |
| Kweka | 2008 | Mosquito abundance, bed net coverage and other factors associated with variations in sporozoite infectivity rates in four villages of rural Tanzania | Tanzania | *Anopheles funestus complex* | Indoors | PSC | 719 | 811 |
| Kweka | 2008 | Mosquito abundance, bed net coverage and other factors associated with variations in sporozoite infectivity rates in four villages of rural Tanzania | Tanzania | *Anopheles arabiensis* | Indoors | PSC | 81 | 727 |
| Kweka | 2008 | Vector species composition and malaria infectivity rates in Mkuzi, Muheza District, north-eastern Tanzania | Tanzania | *Anopheles gambiae* | Indoors | PSC + CDC | 1129 | 1224 |
| Kweka | 2008 | Vector species composition and malaria infectivity rates in Mkuzi, Muheza District, north-eastern Tanzania | Tanzania | *Anopheles funestus complex* | Indoors | PSC + CDC | 251 | 283 |
| Kweka | 2008 | Vector species composition and malaria infectivity rates in Mkuzi, Muheza District, north-eastern Tanzania | Tanzania | *Anopheles funestus complex* | Indoors | PSC + CDC | 51 | 80 |
| Kweka | 2008 | Vector species composition and malaria infectivity rates in Mkuzi, Muheza District, north-eastern Tanzania | Tanzania | *Anopheles funestus complex* | Indoors | PSC + CDC | 33 | 50 |
| Mahande | 2007 | Feeding and resting behaviour of malaria vector, *Anopheles arabiensis* with reference to zooprophylaxis. | Tanzania | *Anopheles arabiensis* | Indoors | PSC | 166 | 417 |
| Mahande | 2007 | Feeding and resting behaviour of malaria vector, *Anopheles arabiensis* with reference to zooprophylaxis. | Tanzania | *Anopheles arabiensis* | Outdoors | Pit traps | 0 | 417 |
| Mahande | 2007 | Feeding and resting behaviour of malaria vector, *Anopheles arabiensis* with reference to zooprophylaxis. | Tanzania | *Anopheles arabiensis* | Indoors | PSC | 291 | 417 |
| Mahande | 2007 | Feeding and resting behaviour of malaria vector, *Anopheles arabiensis* with reference to zooprophylaxis. | Tanzania | *Anopheles arabiensis* | Outdoors | Pit traps | 41 | 417 |
| Muriu | 2007 | Host choice and multiple blood feeding behaviour of malaria vectors and other anophelines in Mwea rice scheme, Kenya | Kenya | *Anopheles arabiensis* | Indoors | PSC | 194 | 2467 |
| Muriu | 2007 | Host choice and multiple blood feeding behaviour of malaria vectors and other anophelines in Mwea rice scheme, Kenya | Kenya | *Anopheles arabiensis* | Outdoors | CDC | 5 | 75 |
| Muriu | 2007 | Host choice and multiple blood feeding behaviour of malaria vectors and other anophelines in Mwea rice scheme, Kenya | Kenya | *Anopheles funestus complex* | Indoors | PSC | 51 | 181 |
| Temu | 2007 | Identification of four members of the *Anopheles funestus* (Diptera: Culicidae) group and their role in *Plasmodium falciparum* transmission in Bagamoyo coastal Tanzania | Tanzania | *Anopheles funestus complex* | Indoors | CDC | 66 | 120 |
| Kent | 2007 | Seasonality, blood feeding behaviour, and transmission of *Plasmodium falciparum* by *Anopheles arabiensis* after an extended drought in southern Zambia. | Zambia | *Anopheles arabiensis* | Indoors | PSC | 415 | 450 |
| Kulkarni | 2006 | Entomological evaluation of malaria vectors at different altitudes in Hai District, Northeastern Tanzania | Tanzania | *Anopheles arabiensis* | Indoors | PSC | 668 | 905 |
| Kulkarni | 2006 | Entomological evaluation of malaria vectors at different altitudes in Hai District, Northeastern Tanzania | Tanzania | *Anopheles arabiensis* | Outdoors | Pit traps | 36 | 144 |
| Kulkarni | 2006 | Entomological evaluation of malaria vectors at different altitudes in Hai District, Northeastern Tanzania | Tanzania | *Anopheles funestus complex* | Indoors | PSC | 57 | 86 |
| Yohannes | 2005 | Can source reduction of mosquito larval habitat reduce malaria transmission in Tigray, Ethiopia? | Ethopia | *Anopheles arabiensis* | Indoors | PSC | 141 | 194 |
| Awolola | 2005 | Identification of three members of the *Anopheles funestus* (Diptera: Culicidae) group and their role in malaria transmission in two ecological zones in Nigeria | Nigeria | *Anopheles funestus complex* | Both | Indoor manual collections +pit traps | 173 | 264 |
| Awolola | 2005 | Identification of three members of the *Anopheles funestus* (Diptera: Culicidae) group and their role in malaria transmission in two ecological zones in Nigeria | Nigeria | *Anopheles funestus complex* | Both | Indoor manual collections +pit traps | 187 | 299 |
| Kamau | 2003 | *Anopheles parensis*: the main member of the *Anopheles funestus* species group found resting inside human dwellings in Mwea area of central Kenya toward the end of the rainy season. | Kenya | *Anopheles funestus complex* | Indoors | Indoor manual collection | 2 | 139 |
| Wanji | 2003 | Anopheles species of the mount Cameroon region: biting habits, feeding behaviour and entomological inoculation rates. | Cameroon | *Anopheles gambiae* | Indoors | PSC | 156 | 235 |
| Wanji | 2003 | Anopheles species of the mount Cameroon region: biting habits, feeding behaviour and entomological inoculation rates. | Cameroon | *Anopheles funestus complex* | Indoors | PSC | 72 | 235 |
| Mwangangi | 2003 | Blood-meal analysis for anopheline mosquitoes sampled along the Kenyan coast. | Kenya | *Anopheles gambiae* | Indoors | PSC | 307 | 338 |
| Mwangangi | 2003 | Blood-meal analysis for anopheline mosquitoes sampled along the Kenyan coast. | Kenya | *Anopheles arabiensis* | Indoors | PSC | 72 | 79 |
| Mwangangi | 2003 | Blood-meal analysis for anopheline mosquitoes sampled along the Kenyan coast. | Kenya | *Anopheles funestus complex* | Indoors | PSC | 378 | 439 |
| Ijumba | 2002 | Malaria transmission risk variations derived from different agricultural practices in an irrigated area of northern Tanzania. | Tanzania | *Anopheles arabiensis* | Indoors | PSC | 380 | 795 |
| Ijumba | 2002 | Malaria transmission risk variations derived from different agricultural practices in an irrigated area of northern Tanzania. | Tanzania | *Anopheles arabiensis* | Indoors | PSC | 132 | 193 |
| Ijumba | 2002 | Malaria transmission risk variations derived from different agricultural practices in an irrigated area of northern Tanzania. | Tanzania | *Anopheles arabiensis* | Indoors | PSC | 160 | 241 |
| Ijumba | 2002 | Malaria transmission risk variations derived from different agricultural practices in an irrigated area of northern Tanzania. | Tanzania | *Anopheles arabiensis* | Outdoors | Pit traps | 21 | 501 |
| Ijumba | 2002 | Malaria transmission risk variations derived from different agricultural practices in an irrigated area of northern Tanzania. | Tanzania | *Anopheles arabiensis* | Outdoors | Pit traps | 44 | 174 |
| Ijumba | 2002 | Malaria transmission risk variations derived from different agricultural practices in an irrigated area of northern Tanzania. | Tanzania | *Anopheles arabiensis* | Outdoors | Pit traps | 4 | 121 |
| Sousa | 2001 | Dogs as a favoured Host Choice of *Anopheles gambiae s.s.* (Diptera: Culicidae) of Sao Tomé, West Africa | Sao Tome | *Anopheles gambiae* | Indoors | CDC | 399 | 434 |
| Sousa | 2001 | Dogs as a favoured Host Choice of *Anopheles gambiae s.s.* (Diptera: Culicidae) of Sao Tomé, West Africa | Sao Tome | *Anopheles gambiae* | Indoors | PSC | 181 | 193 |
| Sousa | 2001 | Dogs as a favoured Host Choice of *Anopheles gambiae s.s.* (Diptera: Culicidae) of Sao Tomé, West Africa | Sao Tome | *Anopheles gambiae* | Outdoors | Indoor manual collection | 113 | 422 |
| Bøgh | 2001 | Effect of Passive Zooprophylaxis on Malaria Transmission in the Gambia | Gambia | *Anopheles gambiae* | Indoors | PSC | 99 | 177 |
| Bøgh | 2001 | Effect of Passive Zooprophylaxis on Malaria Transmission in the Gambia | Gambia | *Anopheles gambiae* | Indoors | PSC | 96 | 185 |
| Habtewold | 2001 | The feeding behaviour and *Plasmodium* infection of *Anopheles* mosquitoes in southern Ethiopia in relation to use of insecticide-treated livestock for malaria control | Ethopia | *Anopheles arabiensis* | Indoors | Indoor manual collection | 27 | 64 |
| Charlwood | 2001 | The impact of indoor residual spraying with malathion on malaria in refugee camps in eastern Sudan | Sudan | *Anopheles arabiensis* | Indoors | PSC | 123 | 242 |
| Magbity | 1997 | Effects of community-wide use of lambdacyhalothrin-impregnated bed nets on malaria vectors in rural Sierra Leone. | Sierra Leone | *Anopheles gambiae* | Indoors | PSC + CDC | 249 | 253 |
| Magbity | 1997 | Effects of community-wide use of lambdacyhalothrin-impregnated bed nets on malaria vectors in rural Sierra Leone. | Sierra Leone | *Anopheles gambiae* | Indoors | PSC + CDC | 397 | 401 |
| Hadis | 1997 | Host choice by indoor-resting *Anopheles arabiensis* in Ethiopia | Ethiopia | *Anopheles arabiensis* | Indoors | Indoor manual collection | 118 | 130 |
| Githeko | 1994 | Origin of blood meals in indoor and outdoor resting malaria vectors in western Kenya | Kenya | *Anopheles arabiensis* | Indoors | PSC | 108 | 232 |
| Githeko | 1994 | Origin of blood meals in indoor and outdoor resting malaria vectors in western Kenya | Kenya | *Anopheles funestus complex* | Indoors | PSC | 86 | 94 |
| Githeko | 1994 | Origin of blood meals in indoor and outdoor resting malaria vectors in western Kenya | Kenya | *Anopheles arabiensis* | Outdoors | Indoor manual collection | 0 | 186 |
| Mbogo | 1993 | Blood feeding behaviour of *Anopheles gambiae s.l*. and *Anopheles funestus* in Kilifi district, Kenya | Kenya | *Anopheles funestus complex* | Indoors | Indoor manual collection | 57 | 64 |
| Garrett-Jones | 1964 | The human blood index of malaria vectors in relation to epidemiological assessment | Multiple | *Anopheles funestus complex* | Indoors | Indoor manual collection | 179 | 183 |
| Garrett-Jones | 1964 | The human blood index of malaria vectors in relation to epidemiological assessment | Multiple | *Anopheles funestus complex* | Outdoors | Indoor manual collection | 61 | 72 |
| Garrett-Jones | 1964 | The human blood index of malaria vectors in relation to epidemiological assessment | Multiple | *Anopheles funestus complex* | Indoors | Indoor manual collection | 1768 | 2056 |
| Garrett-Jones | 1964 | The human blood index of malaria vectors in relation to epidemiological assessment | Multiple | *Anopheles funestus complex* | Outdoors | Indoor manual collection | 110 | 243 |
| Garrett-Jones | 1964 | The human blood index of malaria vectors in relation to epidemiological assessment | Multiple | *Anopheles funestus complex* | Indoors | Indoor manual collection | 99 | 120 |
| Garrett-Jones | 1964 | The human blood index of malaria vectors in relation to epidemiological assessment | Multiple | *Anopheles funestus complex* | Outdoors | Indoor manual collection | 152 | 423 |
| Garrett-Jones | 1964 | The human blood index of malaria vectors in relation to epidemiological assessment | Multiple | *Anopheles funestus complex* | Indoors | Indoor manual collection | 178 | 344 |
| Garrett-Jones | 1964 | The human blood index of malaria vectors in relation to epidemiological assessment | Multiple | *Anopheles funestus complex* | Outdoors | Indoor manual collection | 9 | 193 |
| Garrett-Jones | 1964 | The human blood index of malaria vectors in relation to epidemiological assessment | Cameroon | *Anopheles funestus complex* | Indoors | Indoor manual collection | 53 | 72 |
| Garrett-Jones | 1964 | The human blood index of malaria vectors in relation to epidemiological assessment | Zimbabwe | *Anopheles funestus complex* | Outdoors | Indoor manual collection | 0 | 161 |
| Garrett-Jones | 1964 | The human blood index of malaria vectors in relation to epidemiological assessment | Multiple | *Anopheles gambiae* | Indoors | Indoor manual collection | 258 | 327 |
| Garrett-Jones | 1964 | The human blood index of malaria vectors in relation to epidemiological assessment | Multiple | *Anopheles gambiae* | Outdoors | Indoor manual collection | 3 | 124 |
| Garrett-Jones | 1964 | The human blood index of malaria vectors in relation to epidemiological assessment | Multiple | *Anopheles gambiae* | Indoors | Indoor manual collection | 1844 | 2311 |
| Garrett-Jones | 1964 | The human blood index of malaria vectors in relation to epidemiological assessment | Multiple | *Anopheles gambiae* | Outdoors | Indoor manual collection | 172 | 310 |
| Garrett-Jones | 1964 | The human blood index of malaria vectors in relation to epidemiological assessment | Multiple | *Anopheles gambiae* | Indoors | Indoor manual collection | 418 | 537 |
| Garrett-Jones | 1964 | The human blood index of malaria vectors in relation to epidemiological assessment | Multiple | *Anopheles gambiae* | Outdoors | Indoor manual collection | 147 | 524 |
| Garrett-Jones | 1964 | The human blood index of malaria vectors in relation to epidemiological assessment | Multiple | *Anopheles gambiae* | Indoors | Indoor manual collection | 566 | 696 |
| Garrett-Jones | 1964 | The human blood index of malaria vectors in relation to epidemiological assessment | Multiple | *Anopheles gambiae* | Outdoors | Indoor manual collection | 29 | 964 |
| Garrett-Jones | 1964 | The human blood index of malaria vectors in relation to epidemiological assessment | Multiple | *Anopheles gambiae* | Indoors | Indoor manual collection | 16 | 1114 |
| Garrett-Jones | 1964 | The human blood index of malaria vectors in relation to epidemiological assessment | Multiple | *Anopheles gambiae* | Outdoors | Indoor manual collection | 300 | 940 |
| Garrett-Jones | 1964 | The human blood index of malaria vectors in relation to epidemiological assessment | Multiple | *Anopheles gambiae* | Indoors | Indoor manual collection | 196 | 265 |
| Garrett-Jones | 1964 | The human blood index of malaria vectors in relation to epidemiological assessment | Multiple | *Anopheles gambiae* | Outdoors | Indoor manual collection | 99 | 226 |
